# Fundamental Methods for the Phase Transfer of Nanoparticles

**DOI:** 10.3390/molecules26206170

**Published:** 2021-10-13

**Authors:** Elijah Cook, Gianna Labiento, Bhanu P. S. Chauhan

**Affiliations:** Engineered Nanomaterials Laboratory, Department of Chemistry, William Paterson University, 300 Pompton Road, Wayne, NJ 07470-2103, USA; cooke7@student.wpunj.edu (E.C.); labientog@student.wpunj.edu (G.L.)

**Keywords:** nanoparticles, phase transfer, ligands, gold, organic solvents, drug delivery, catalysis, thermoregulation, host–guest interaction, interdigitated layers

## Abstract

The utilization of nanoparticles for a variety of applications has raised much interest in recent years as new knowledge has emerged in nanochemistry. New and diverse methods for synthesis, characterization, and application of these particles have been discovered with differing degrees of ease and reproducibility. Post-synthetic modification of nanoparticles is often a required step to facilitate their use in applications. The reaction conditions and chemical environment for the nanoparticle synthesis may not support or may conflict with further reactions. For this reason, it is beneficial to have phase transfer methods for nanoparticles to allow for their dispersion in a variety of solvents. Phase transfer methods are often limited in the types and sizes of particles that can be effectively dispersed in an immiscible solvent. Currently, general transfer methods for a wide variety of nanoparticles have not been identified. New routes for phase transfer allow for utilization of a larger range of particles in applications which were previously limited by solubility and reactivity issues. In this work, we will describe the fundamental methods for the phase transfer of metallic nanoparticles. We will look at the major problems and pitfalls of these methods. The applications of phase transfer will also be reviewed, mainly focusing on catalysis and drug delivery.

## 1. Introduction

Nanoparticles are an area of intense interest due to their unique physical, chemical, and biological properties, which make them useful in many applications. In the last 30 years, extensive research has been conducted which has provided a plethora of routes and methods for the synthesis of nanoparticles with different shapes, sizes, and solubility [1]. It has been observed that subtle changes in reaction conditions, precursors, or stabilizing agents can endow nanoparticles with specific and desired properties [1]. However, limitations such as solubility and polarity have also been commonly observed. Since many synthetic methods rely on the use of specific hydrophobic or hydrophilic interactions to stabilize the particles, the resulting materials will have those corresponding polarities [1].

For instance, biological applications require nanoparticles to be water dispersible and remain soluble [2]. On the other hand, many catalytic and/or industrial applications require nanoparticles to be dispersed in organic solvents [3,4]. Synthetic strategies which utilize aqueous or organic media have their advantages and disadvantages based on the application of the resulting nanomaterial. Aqueous nanoparticle synthesis can be fraught with problems. For example, aqueous synthetic routes suffer from difficulties in separating excess stabilizing agents due to strong ionic interactions [5]. However, there are many well-defined methods to produce nanoparticles in aqueous solution, as they can solubilize and stabilize many precursor ions and molecules. In general, ionic interactions present in aqueous solutions cause nanoparticles to be synthesized at lower concentrations than in organic solvents [4,5,6,7]. On the other hand, the solubility of common metal ions is limited in organic solvents. Therefore, one must tailor precursors in order to reduce them in organic media.

Based on the synthetic methods, many desired transformations cannot be carried out due to solubility barriers keeping the particles in separate phases. Traditionally, this issue has been resolved by using solvents such as DMSO, which can at least partially solubilize materials in both media. Phase transfer can be a strategy which can provide a solution to such problems. It can be a useful tool to facilitate reactions that were previously inaccessible, due to phase separation.

Phase transfer is the physical phenomenon where particles or molecules are transported between two immiscible solvents. In order to transfer particles from one solvent to another, the chemical properties must be altered in a way that allows for stable, low-energy interaction between particles and new solvent molecules. This strategy permits many reactions which otherwise face a solubility barrier to be carried out. The phase transfer agent must be able to transfer the particles in an active state so that the reaction can proceed.

Phase transfer plays an important role in facilitating reactions where the reactants exist in two immiscible phases, such as when nanoparticles (NPs)are to be enclosed in an organic matrix as in the case of applications involving molecular electronics [8]. Creating methods to transfer nanoparticles between aqueous and organic phases is highly desired because this would provide a way to utilize a variety of synthetic methods and nanoparticle types for applications in different chemical systems. The following examples in this review demonstrate the utility of this approach.

There are quite a few well-defined protocols for synthesizing gold nanoparticles in aqueous media [1]. However, the catalytic applications of gold nanoparticles require them to be dispersible in organic solvents [3]. Though one can devise a strategy to produce similar materials in organic media, they might not possess the same catalytic properties as those produced in aqueous media. Instead of synthesizing the particles in the solvent of interest, it may be beneficial to phase transfer the particles from the optimal solvent for synthesis to one tailored for the process being studied.

For many reactions and applications, prepared nanoparticles require post-synthetic modifications to tailor their solubility, stability, biocompatibility, as well as a variety of other properties [6]. Surface modification has been the method of choice to tailor nanoparticles’ chemical, physical, and biological properties [1]. Since interparticle interactions play a very important role, any small change in stabilizing molecules will affect the stability, the solubility, and the application of the nanoparticles [6,9].

Known methods in the literature applying phase transfer techniques seem to be only attuned with nanoparticles of a certain size and shape [4]. In addition, it has been observed that certain phase transfer protocols are not easily reproducible, and complicated procedures have a high probability for side reactions [4]. In many methods, while attempting the phase transfer of nanoparticles, the surface properties of nanoparticles are altered permanently [7]. In summary, the development of new strategies for phase transfer is required where such barriers and solubility issues are avoided.

The end goal of phase transfer is to allow for any type of nanoparticles to be dispersed in both hydrophobic and hydrophilic solvents, which maintain all the chemical properties and characteristics of the original nanoparticles. There are two approaches to the phase transfer of nanoparticles, with the first being sedimentation/redispersion, and the second phase transfer through the liquid–liquid interface [10]. Thus, stabilizing agents present on the nanoparticle surface will affect which phase transfer methods are compatible for that particular system. For example, some transfer methods require a pre-stabilization step to coat the nanoparticles with a specific ligand for phase transfer to be successful.

As is well understood, stabilizing ligands affect the properties of nanoparticles in a very significant fashion. Solubility, stability, and functional compatibility are affected by the choice of ligand on the nanoparticle surface. Commonly, long-chain organic molecules containing hetero-atoms, polymers, dendrimers, and functionalized biomolecules have been used to prepare nanoparticles [10,11,12,13]. Such molecules which are bound to the surface of the nanoparticles are generally referred to as ligands, surfactants, or capping agents. Surface ligands on a nanoparticle can offer multiple opportunities to influence the properties of nanomaterials. For example, drug delivery applications require nanoparticles to be able to both carry cargo/drug and target the active site [14]. Multifunctional surfactants, or a combination of surfactants, are required to be present on the nanoparticle to be relevant for this application [14,15]. The structure, the stability, and the function of the nanoparticle are dependent on the ligand, which allows one to use post-synthetic ligand modification for further applications of nanoparticles. While performing such modifications, attention should be paid to preserve the original properties of the particles if they are desired after the modification of nanoparticles.

The bonding affinity of a stabilizing agent alters the surface properties of the nanoparticle as well. For example, thiols have a high affinity for gold and silver, which makes them useful as surface ligands for stabilizing the corresponding nanoparticles. Due to this strong bonding, the overall stability of the nanoparticles is also influenced, and the particles remain stable in solutions for longer periods of time. On the other hand, ligands with low affinity for the particle surface will produce less stable structures and will lead to agglomeration/precipitation over time. It has been observed that nanoparticle stability can be increased by utilizing multidentate ligands [14].

The nanoparticle environment, whether organic or aqueous, is a consequence of the solubility of the surface ligand [14]. Nanoparticles may need to be dispersed in different organic media, buffers, and solvents with varying pHs. For applications in biological systems, biocompatible ligands must be utilized to prevent degradation of the nanoparticles [14]. Ligands are also chosen to create specific conditions for biological targeting.

It is well documented that the size and the shape of nanoparticles are strongly influenced by the ligand system used for their stabilization. Consequently, characteristics such as packing characteristics, diffusion rates, and binding energies play a very important role in creating new morphologies of nanoparticles [14]. Ligands with high affinity for the particles, such as thiols and gold, will often produce smaller particles with stronger particle–ligand bonding. On the other hand, those with weaker binding affinity, such as citrate and gold, allow for the creation of larger particles.

Post-synthetic modification of the ligand shell with additional biomolecules or specific polymers is often required to provide desired properties for biological applications. The potential functionalities that can be added are limited by the interactions at the particle surface.

By providing more reactive or selective ligands, the catalytic properties of nanoparticles can be influenced. A problem with certain capping ligands is the potential for them to act as a physical barrier restricting the access of reactants to the nanoparticle surface [14]. In addition, the surface ligand can also react with the substrates undergoing catalysis and consume the reactants while breaking down the structure of nanoparticles. That can have a detrimental effect on the catalytic properties of nanoparticles. Due to these issues, it is imperative that the surfactant on the surface of the nanoparticles be carefully chosen so that they can beneficially affect the catalytic properties of the nanoobjects.

## 2. Major Techniques for Phase Transfer

**Figure 1:**
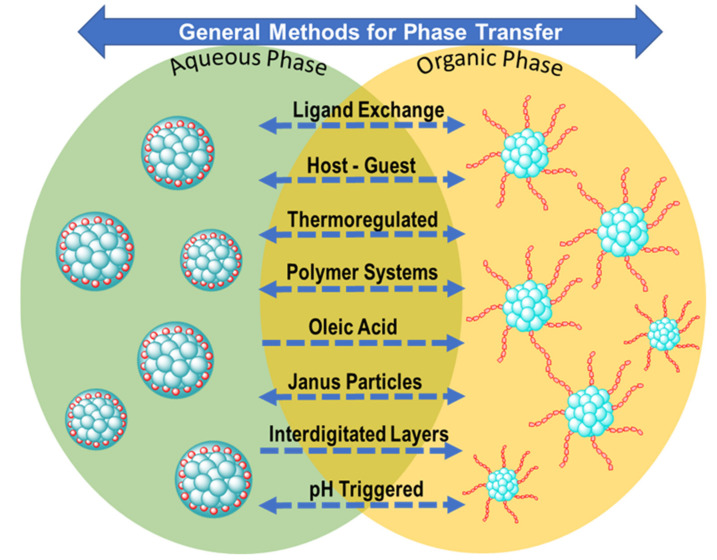
Schematic representation of the general methods for the phase transfer of nanoparticles.

Nanoparticle solubility relies on the interaction between the solvent and the stabilizing agent. Through modification of the capping agents, the hydrophilicity or hydrophobicity can be controlled. Modification to attach ligands is carried out in a controlled fashion which permits the nanoparticles to retain their original properties and change their solubility behavior. Figure 1 summarizes the general known methods for phase transfer. In many applications, the post-synthetic phase transfer of nanoparticles between immiscible solvents is required. In such cases, ligand exchange or substitution has been used as the methodology of choice [1]. In the following sections, major techniques for phase transfer are summarized.

### 2.1. Ligand Exchange

**Figure 2:**
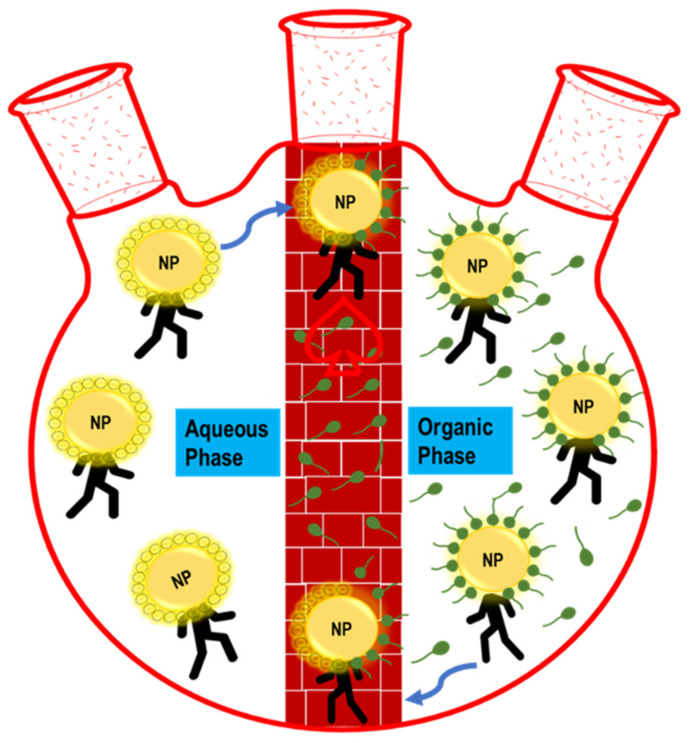
Schematic representation of phase transfer via a ligand-exchange reaction. In the figure, the abbreviation “NP” means nanoparticle.

One method to achieve a change in stabilizing agent is through ligand-exchange reactions. This substitution reaction facilitates the removal of the attached ligands and their replacement with new ligands, tailored for specific chemical environments and applications (see Figure 2 below) [1]. Replacing a hydrophilic capping ligand with one that offers hydrophobic properties can promote the phase transfer of the particles by changing the way the particle surface interacts with the solvent molecules. Ligand-exchange reactions can affect nanoparticle characteristics and must be designed to retain the original chemical properties and reactivity [16]. These ligand-exchange reactions are often completed in biphasic mixtures, allowing for phase transfer of colloidal solutions to a new solvent system. New surface ligands on nanoparticles will affect their overall size and shape, so important considerations must be made when identifying a new ligand.

Ligand-exchange reactions can be useful in protecting nanoparticles from harsh chemical environments, thereby maximizing their properties for biological systems [2]. Potential harsh conditions in the body, such as extreme pH, can degrade particles. The design of surface molecules will greatly influence the protection and biodistribution of nanoparticles in biological systems [2]. Ligand-exchange reactions completely change the stabilizing agent, thereby removing unwanted properties and providing a new environment for the particles.

#### 2.1.1. Alkanethiols

Many methods dating back years describe the use of alkanethiols in the phase transfer of nanoparticles. Giersig and Mulvaney first reported the use of alkanethiols for the phase transfer of gold particles into organic solvents in 1993 [17]. They demonstrated that thiols with varying chain length are very desirable for transfer of gold particles as they form an extremely strong bond to the particle surface. This would provide tailored nanoparticles which can be utilized in various chemical environments. It has been shown that the ligand exchange allows for control over the nanoparticle core size and composition, as well as the chemical nature of the stabilizing shell [15].

Alkyl chain length, along with the concentration of the thiols, has an important role in the stabilization of nanoparticles in a new environment [18]. Thiolated molecules have also proved useful for phase transfer of other metallic nanoparticles. In one instance, thiolated polyethylene glycol (PEG) was used along with a hydrophobic stabilizer to transfer gold particles [19]. It has also been shown that different steric functionalizations on the alkylthiols affect the efficiency of phase transfer [18]. In the case comparing dimethyldodecanethiol with octadecanethiol, the two methyl groups on the former cause some steric strain between the ligands [18]. The octadecanethiol has its sulfur surrounded by hydrogens, and therefore the particles have a more efficient transfer. Longer alkyl chains have been found to require longer times for transfer [18]. Not only can alkanethiols act as transfer agents, but they can also stabilize nanoparticles in organic solvents for prolonged storage.

#### 2.1.2. Alkylamines

Alkylamines are very commonly utilized as phase transfer agents and in ligand-exchange reactions. Many detailed protocols have been published describing the conditions needed for transfer [20,21,22,23,24,25,26]. In one example dimethylaminopyridine was used to transfer gold and palladium particles across the phase boundary into water without any stirring or agitation [5]. As the particles were synthesized in organic solution, their concentration was much higher than a complimentary aqueous synthesis.

Like alkanethiols, alkyl chain length on alkylamines plays a major role in influencing the effectiveness of the transfer. In a study on the effects of chain length, three amines of different lengths were used for a surface modification [8]. The ligands were added in a mixture of toluene and acetone. The acetone helps to break the surface tension between the two phases. It was shown that increasing chain length often helps to stabilize the transferred particles [8]. Shorter alkyl chains showed the ability to transfer particles but failed to stabilize them in toluene [8]. The reagents used for the initial particle synthesis also affect the availability of the particle surface for further functionalization [5,8].

Ligand exchange using a bifunctional α-trithiocarbonate-ω-carboxyl-terminated poly(*N*-isopropylacrylamide) (PNIPAM) ligand allowed for reversable phase transfer through temperature and pH [13]. This was carried out through the terminal trithiocarbonate group, which also provides an anchor point for further chemical modification [13].

#### 2.1.3. Alkylphosphonic Acids

Phosphonic acids are similar in structure to alkylthiols and amines. However, they do not polycondense through P–O–P bonds and are stable in aqueous medium, making them interesting phase transfer agents [10]. It was shown that phosphonium-stabilized gold nanoparticles are stable in both water and DMSO [10].

Phosphonic acids are particularly useful in organic-to-aqueous phase transfer of metal oxide particles. It has been proposed that the stabilizing metal oxide–phosphonic acid bonds have greater stability than those formed by carboxylic acids, sulfates, or amines [10]. A dense film of alkyl chains is often required to facilitate the transfer of nanoparticles. It has been shown that the dispersion of transferred nanoparticles is dependent on the properties of the phosphonic acid, as longer alkyl chains supported the best dispersion [10]. 

### 2.2. Phase Transfer via Chemical Modification with Oleic Acid Ligands

Oleic acid can be both added or replaced in a ligand exchange to facilitate phase transfer. Published protocols describe the synthesis of oleic acid-stabilized metallic nanoparticles [27]. Oleic acid-stabilized Ag nanoparticles can be phase transferred to aqueous solution through ligand exchange with cyclodextrin [27]. The efficiency of this phase transfer was dependent on the cyclodextrin concentration, as it must effectively replace the oleic acid ligands [27]. In this instance, oleic acid is being replaced to facilitate the phase transfer.

**Figure 3:**
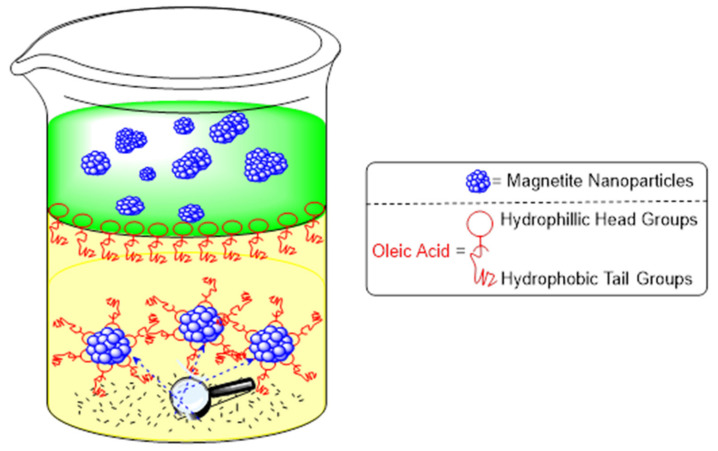
Schematic representation of phase transfer via modification with oleic acid ligands. The scheme is derived from [28].

Oleic acid also holds the ability to transfer nanoparticles by sedimentation through the liquid–liquid interface (see Figure 3 below) [28]. The particles are first destabilized and allowed to agglomerate. As sedimentation begins to occur, the particles fall through the liquid–liquid interface, which is lined with many surfactant molecules. This adsorption of oleic acid onto the particles leads to the disintegration of the aggregates and allows for dispersion of the particles in organosols [28].

Another method of transfer for oleic acid is through oxidative cleavage and formation of reverse micelles [29]. Oleic acid-stabilized particles can be oxidatively cleaved to endow the nanoparticle surface with hydrophilic properties [29]. Once full oxidation is complete, the nanoparticles are stabilized by hydrophilic molecules containing carboxyl groups. These transferred particles, stabilized by oleic acid, showed good aqueous dispersibility, stability, and low cell cytotoxicity [29]. Furthermore, the terminal carboxyl groups can be additionally tailored through functionalization with drug molecules or active biomolecules [29].

### 2.3. Phase Transfer via Polymer-Based Systems

Polymer-stabilized nanoparticles are often used in biological applications as they can produce stable particles with low cell cytotoxicity [2]. The phase transfer of nanoparticles through the use of polymers provides a few different possible methods [4,30,31,32].

In one example, poly(*N*-vinylpyrrolidone) PVP-stabilized metal nanoparticles were phase transferred from aqueous to organic phases through change in temperature of oil-water mixtures [4]. When heat is applied to the water–butanol mixtures, the hydrogen bonds between PVP and water are broken, and the solubility of the nanoparticles decreases. As these bonds are destroyed, a co-stabilizer forms a polymer–surfactant complex, which increases the hydrophobicity of the particles [4]. This method allowed for efficient phase transfer of gold, silver, and platinum nanoparticles from the aqueous phase to the butanol phase [4]. The ratio between PVP and the metal salt during synthesis dictates the resulting shapes and sizes of the particles [4].

**Figure 4:**
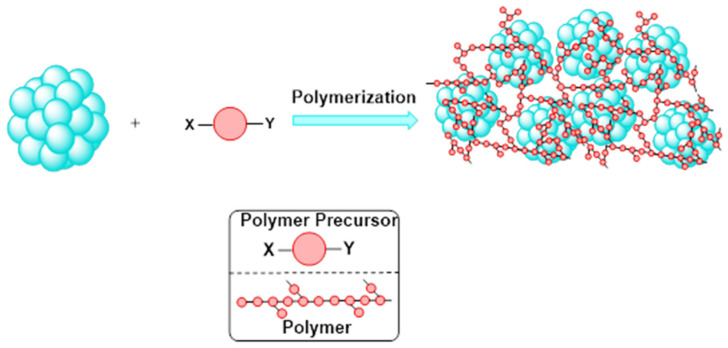
Schematic representation of phase transfer through polymer-based systems [2].

The polymer wrapping method is another useful way to transfer nanoparticles between two immiscible phases (see Figure 4 below). Polymer wrapping involves wrapping nanoparticles synthesized in organic solvents with amphiphilic polymers to stabilize them in aqueous solution [2]. The protocol by Del Pino et al. describes an extension of this method wherein nanoparticles synthesized in aqueous media can be transferred in a 2-step method [2]. This phase transfer method was demonstrated with Au and Ag nanoparticles of different shapes and sizes up to 100 nm [2]. This allows for nanoparticles with different cores, and therefore different physicochemical properties, to have very similar surface chemistry for use in similar applications [2]. The biological compatibility of the nanoparticles is highly dependent on their surface chemistry [33]. The chemical composition of the nanoparticle coating can affect many biological properties such as cellular uptake, nanoparticle localization in cells, toxicity, circulation, biodistribution, protein corona, etc. [2]. This method also produces nanoparticles with high colloidal stability against high salt concentrations and a broad pH range [2]. In this procedure, nanoparticles were first synthesized and then pre-stabilized through a phase transfer to organic media by PEGylation with dodecylamine [2]. The nanoparticles were then water transferred through the polymer wrapping technique. This is carried out through the use of poly(isobutylene-*alt*-maleic-anhydride)(PMA) modified with dodecylamine. After polymer addition, the solvent was evaporated, and the nanoparticles were purified and then redispersed in water. The particle surface was then modified with PEG-NH_2_. The carboxyl groups on PMA and amine groups on the PEG-NH_2_ become cross linked to stabilize the particles. The stability of the particles was tested in common biological systems which indicated their potential for use in biological applications [2].

### 2.4. Phase Transfer via pH Modification

**Figure 5:**
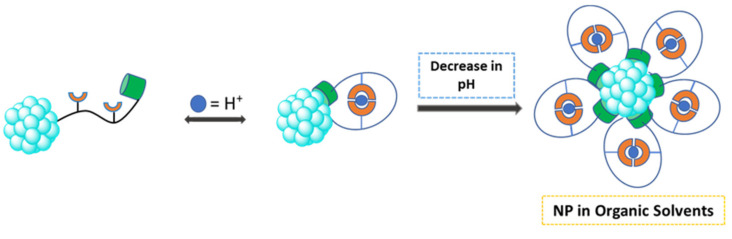
Schematic representation of phase transfer via pH modification [3]. In the figure, the abbreviation “NP” means nanoparticle.

Using changes in pH to transfer nanoparticles between two immiscible solvents is a relatively new method. In one example, thiolated poly(acrylic acid)(PAA-SH) functionalized nanoparticles showed the ability to transfer upon changes in pH [3]. The modified PAA ligands covalently bond to the particle surface through the thiol functional groups. These particles displayed pH responsiveness where aggregation and dispersion was induced at acidic and basic pHs [3]. Adjustment of solution pH and vigorous stirring allowed for reversible phase transfer of the particles from aqueous to organic solvents [3]. This method is extremely useful for the recovery and reuse of catalytic metal nanoparticles. The phase transfer of these particles was carried out without any loss in catalytic activity [3]. Phase transfer using pH allows for solvent exchange to take place without the need for changing the stabilizing agent or the reactivity of the particles [34]. Figure 5, below, shows a potential design for a pH-triggered phase transfer system.

### 2.5. Phase Transfer via Host–Guest Chemistry

As is seen in previous sections, not all phase transfer methods are reversible. The host–guest phase transfer offers a fully reversible method for transfer of particles between immiscible phases [35]. In certain host–guest methods, light is used for the stimuli response for phase transfer and has its own advantages. For example, light can be delivered in a quantitative manner within a precise space and time [35,36].

**Figure 6:**
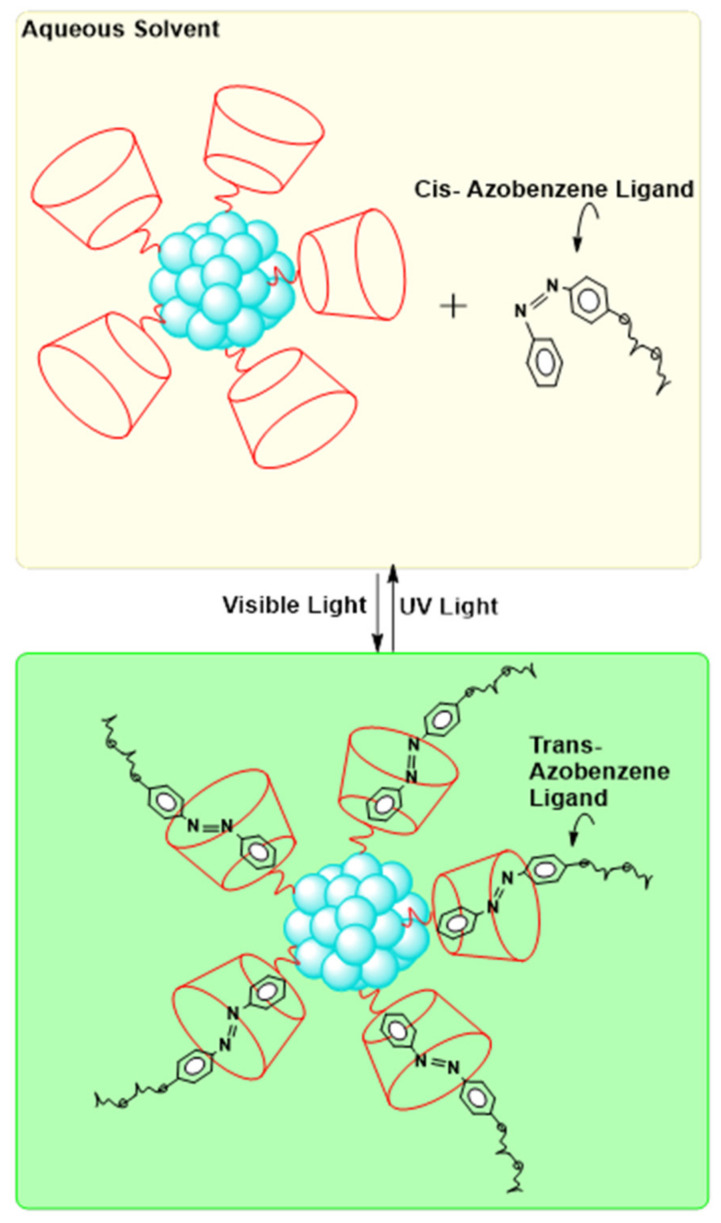
Schematic representation of phase transfer via host–guest chemistry. Scheme derived from [35].

In one report, a photoreversible phase transfer system consisted of an azo-ligand and a thiolated α-cyclodextrine(α-CD) (see Figure 6 below) [35]. Azobenzenes are a class of compounds which undergo photoresponsive cis-trans isomerization using UV and visible light. In this case, the trans azobenzene isomer can form a complex with α-CD through steric and hydrophobic interactions [35]. The cis isomer changes conformation and cannot form the complex [35]. The α-CD AuNPs are first dispersed in water with the trans azo-ligand dispersed in toluene. Through agitation of the biphasic system, the trans azo-ligands are brought into the vicinity of the nanoparticles to form a stable complex, which pulls them into the toluene layer [35]. To reverse the process, one can use UV light [35]. UV light causes the azo-ligand to isomerize from trans to cis, which can no longer form a complex with the α-CD AuNPs. The NPs revert to their hydrophilic nature and return to the water. Exposure to visible light converts the azo-ligand back to its trans form, allowing for the process to be repeated. This method has been applied to quench a nanoparticle catalyzed reaction and to recover and recycle the particles [35]. Host–guest phase transfer, similarly to pH-triggered phase transfer, is highly desired as it provides a method for the fully reversible phase transfer of particles without changing the capping agent.

### 2.6. Phase Transfer via Surface bound Interdigitated Layers

**Figure 7:**
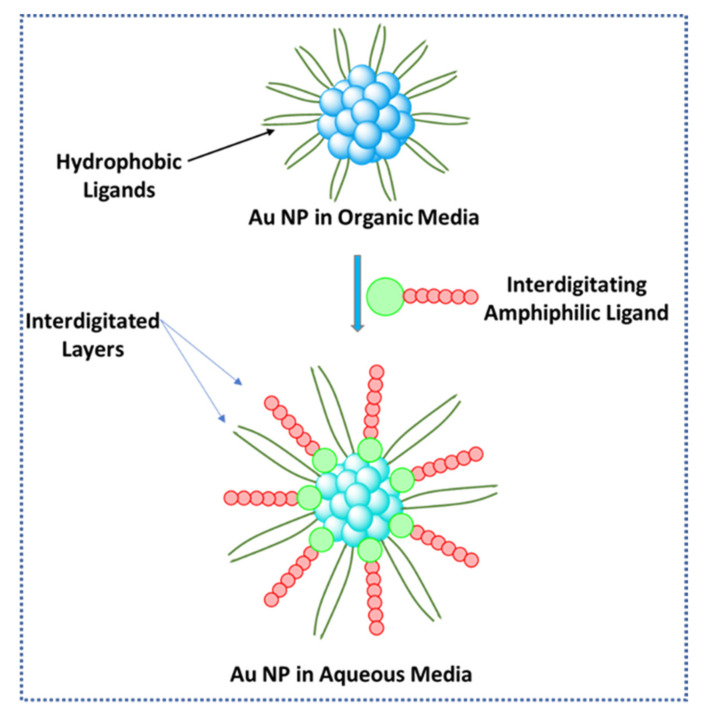
Schematic representation of phase transfer via surface-bound interdigitated layers [7]. Scheme derived from [7]. In the figure, the abbreviation “NP” means nanoparticle.

The use of surface-bound interdigitated layers can provide enough stabilizing forces to transfer nanoparticles to different solvents [7,20]. The electrostatic interaction formed between two different ligands allows the particles to be dispersed into an immiscible solvent [7]. In one example, dodecylamine (DDA) gold nanoparticles were transferred from organic to aqueous media through the use of a cationic surfactant [7]. These transferred particles can be dried and redispersed in water [7]. This route could provide a way to achieve high nanoparticle concentration in water, which is useful for biological applications [7]. Analysis of transferred nanoparticles showed interdigitated bilayers of a DDA primary layer coordinated to the surface of the particle as well as a second monolayer of cetyltrimethylammonium bromide (CTAB) [7]. The CTAB secondary monolayer provides the particles with hydrophilic properties, allowing for their dispersion in aqueous media [7]. The structure of the interdigitated layers is stable because of the hydrophobic interactions between them [7]. In Figure 7, this concept of phase transfer is depicted where interdigitated layers are formed as described above.

Another added advantage of this method is that due to bilayer capping of NPs, direct interaction between solvent and the particle is circumvented [7]. The surface plasmon resonance of these gold particles is not shifted as much as other methods because it is well known that the surface plasmon resonance of gold nanoparticles red-shifts as the refractive index of the solvent media increases [37].

### 2.7. Phase Transfer via Janus Particles

**Figure 8:**
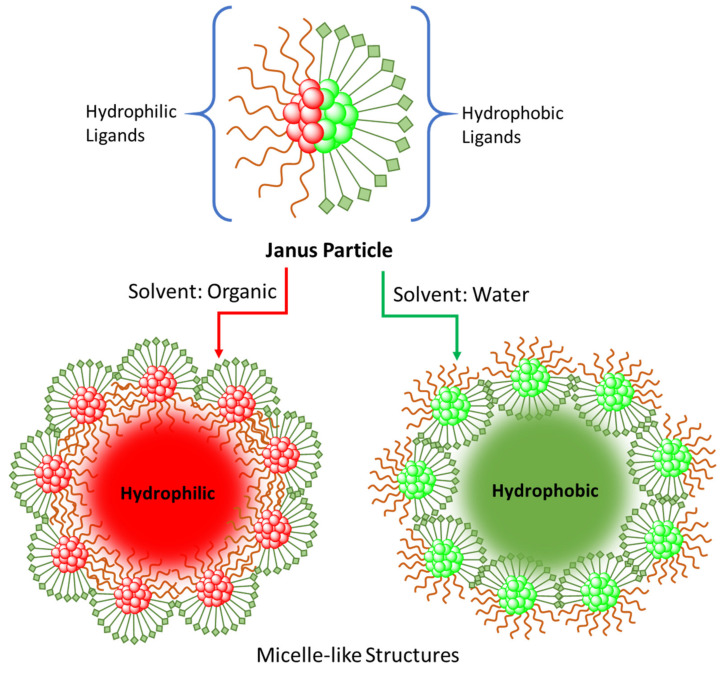
Schematic representation of phase transfer via Janus nanoparticles [38]. Scheme derived from work described in [38].

Janus particles are a unique type of nanoparticles with asymmetrical properties. The asymmetric properties are created using capping agents on the metal nanoparticle surface. For example, the ligands can be grouped on the particle surface forming a hydrophilic and hydrophobic zone, thus creating a Janus nature in the nanoparticle. It has been demonstrated that hydrophobic alkanethiolate protected gold nanoparticles, which are generated at the air–water interface, will undergo an interfacial ligand-exchange reaction with thiol ligands which are hydrophilic in nature, yielding Janus nanoparticles [38] (see Figure 8 below). For example, in work by Chen and coworkers, the hydrophobic 1-hexanethiolate stabilized one side of the particles and hydrophilic 1,2-mercapto-3-propanediol stabilized the other [38]. The asymmetric properties of these nanoparticles allow them to act as surfactants, forming micelle-like structures in both water and organic solvents alike. The particles showed the ability to be transferred from DCM to water and remain stable [38]. They can also act as nanocarriers for the phase transfer of guest nanoparticles or molecules utilizing the inner part of the micelle. These Janus particles acted as phase transfer catalysts for silver nanoparticles and fullerene (C60) molecules [38]. This versatility of Janus particles makes them very interesting agents for future phase transfer applications.

### 2.8. Phase Transfer via Thermoregulated Process

**Figure 9:**
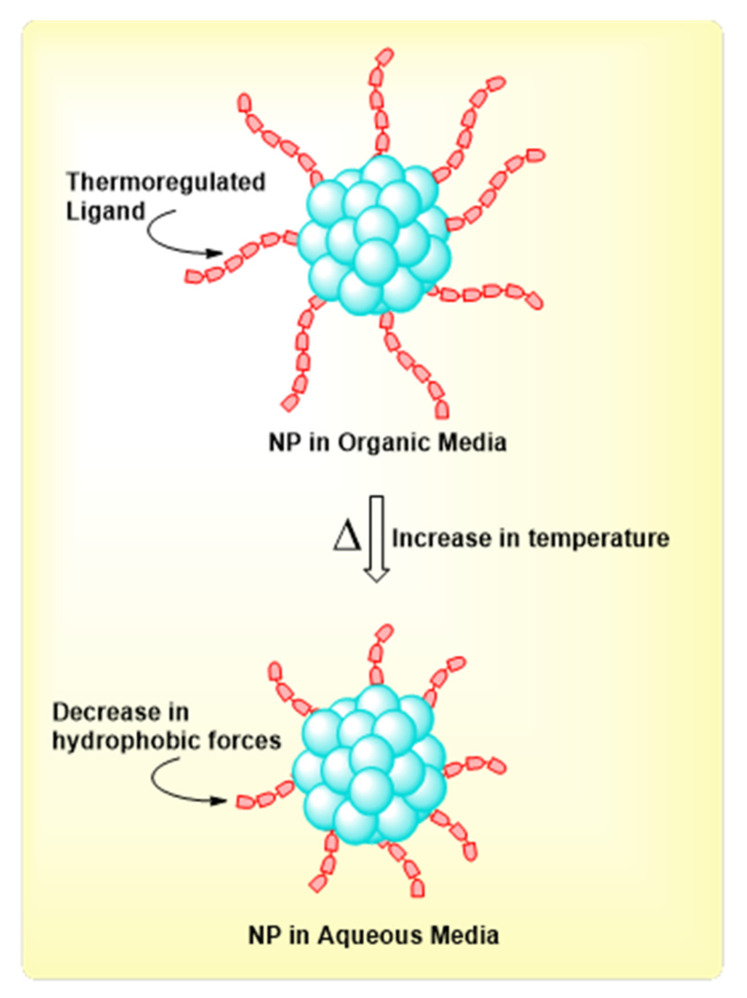
Schematic representation of phase transfer via thermoregulation. In the figure, the abbreviation “NP” means nanoparticle.

Thermoregulated ligands and molecules experience temperature-dependent solubility in water, which allows for separation upon heating. This property can be utilized to support the transfer of particles between solvents through stabilization of a thermoregulated ligand. In these cases, the properties of the ligand are such that the temperature changes allow for the manipulation of the hydrophobic and hydrophilic forces in organic and aqueous media. A cartoon representation of this concept is shown below in Figure 9.

An excellent example of this concept was demonstrated by Qin and coworkers [39]. In this work, they reported the use of 2-(diethylamino)ethanethiol (DEAET) molecules to achieve thermoregulated phase transfer of CdTe nanoparticles [39]. The amphiphilic nature of the free ends of DEAET allow this process to be thermally controlled [39]. At 0 °C the particles precipitate out of water; however, they dissolve and disperse at higher temperatures. The total hydrophobic forces of the ligand decrease with increasing temperature, allowing for dispersion in aqueous media [39]. This process is quick, reversible, and can provide partial transfer if needed. The inter-nanoparticle forces are affected by changes in temperature which causes the particles to aggregate at the interface and then phase transfer into an immiscible solvent [39]. Thermoregulated methods provide a useful alternative for recovery and reuse of catalytic nanoparticles without significant changes in chemical or physical properties [39,40].

It is beneficial to create a library of phase transfer methods such as that which can be found in the literature for the synthesis of nanoparticles. For this reason, Table 1, below, shows details of selected protocols for the phase transfer of nanoparticles which are reviewed in this publication.

## 3. Analytical Tools to Monitor the Phase Transfer Process

Nanoparticles can be analyzed and characterized in two ways: direct and indirect. Direct techniques involve imaging particles in order to directly measure sizes as well as observe shape information. Direct analysis utilizes analytical tools such as transmission electron microscopy (TEM), scanning electron microscopy (SEM), and atomic force microscopy (AFM). These techniques are often limited to analysis of a few particles from a larger sample at a time [57]. Sample preparation for electron microscopy may also bring about issues. These direct methods of analysis are effective for obtaining general information about nanoparticles. Indirect techniques involve using electromagnetic radiation to gain information about the properties of nanoparticles. These methods often require mathematical analysis of the scattered or diffracted waves by the nanoparticles [57]. Some analytical tools used for indirect analysis include UV–VIS spectroscopy, infrared spectroscopy, Raman spectroscopy, thermogravimetric analysis (TGA), nuclear magnetic resonance (NMR), and X-ray diffraction analysis. These methods do not require in-depth sample preparation and can generally analyze and average highly concentrated samples of nanoparticles. The centrifugation of colloidal nanoparticle solutions to isolate the particles is useful for enabling different characterization methods [58].

UV–VIS spectroscopy can be useful for monitoring the phase transfer process of nanoparticles. In previous work from Feng et al., phase transfer of gold nanoparticles from aqueous to organic solution was monitored [4]. This is a way to measure the phase transfer efficiency indirectly through depletion percentage. The UV–VIS spectrum of the aqueous layer before transfer showed a symmetric absorption band at 536 nm [4]. After the transfer process, the aqueous phase gave a very weak absorption at the same wavelength indicating effective transfer. This method allows for the visualization of the concentration of nanoparticles transferred to new media. The phase transfer efficiency for a certain reaction can be mathematically determined using information from the absorbance and the particle concentration [57]. UV–VIS spectroscopy can also provide a comparison of phase transfer methods or ligands for a certain system [59].

Nuclear magnetic resonance (NMR) can quantify the extent of ligand exchange in phase transfer reactions. It can provide information on the ligand-exchange mechanism as well as the ligand density on the nanoparticle surface [60]. Proton NMR looks at the number of hydrogen atoms on a carbon-based molecule and allows for the determination of the structure through splitting and intensity of the spectra. NMR can provide information including the size and surface area of the particle, as well as the chirality of nanoclusters [54]. NMR is also a powerful tool for the analysis of the density, structure, and chemical nature of binding ligands [54]. In phase transfer reactions, it can be applied to determine whether full ligand exchange has proceeded and identify the structure of bound ligands.

TEM images can also provide useful comparison between pre-transfer and post-transfer nanoparticles. Observations such as changes in particle size, shape, or the particle number per unit area may be important in determining mechanisms of transfer [54]. Programs such as ImageJ from the NIH are useful when used in conjunction with TEM images to determine sizes of nanoparticles. Through this post-analysis program, average nanoparticle sizes of different samples can be determined with no need for additional instrumentation. TEM imaging is often limited to smaller sample sizes compared to SEM; however, the high resolution of imaging makes it a desirable for analysis at nanoscale. Comparison of TEM images from pre- and post-transfer are often helpful for identification of morphology, size, or concentration changes.

Fourier transform infrared spectroscopy (FTIR) can be a very useful tool for the analysis of pre- and post-phase transferred nanoparticles. FT-IR allows for identification of the functional groups present in a system through the measurement of the vibrational frequencies of the chemical bonds. This provides a method to monitor and investigate functional groups adsorbed to the nanoparticle surface. Information on the structural and conformational changes of coordinated functional groups at the particle surface can be obtained with this technique [54]. New or changing ligands on the nanoparticle surface can be identified through their vibrational signature in a quick and effective manner. FT-IR is particularly useful in the analysis of ligand-exchange reactions, being able to identify complete exchange.

Raman spectroscopy is an analysis method complimentary to FT-IR. It can also be used to identify and characterize ligands on the nanoparticle surface.

Thermogravimetric analysis is a method where the mass of a substance is monitored as a function of time in a temperature-controlled system. The presence of surface coatings and the purity of nanoparticles can be measured [57]. Nanoparticles with different surface ligands will show different TGA curves. This method is helpful in determining the number of surface-bound ligands on the nanoparticle surface [57]. TGA provides a quantitative determination of ligands at the surface of the particles as well as compositional data on nanoparticles [57].

X-ray diffraction (XRD) is a valuable analytical tool that provides information on the particle size, purity, and sometimes morphology [57]. This method provides information that is complimentary to that of microscopic methods. This allows for comparison between analytical results from a small sample size versus a large sample size. Information on the structure, shape, size, and crystallinity are useful when preforming nanomaterial analysis [57,58].

Each analytical tool has its capabilities and limitations. Combining these methods allows for a full picture of the structure and properties of many different types of nanomaterials.

## 4. Major Pitfalls

Each method for the phase transfer of nanoparticles has its advantages and disadvantages. Many established protocols can only support particles of a certain size and material for efficient transfer. Many early methods utilizing alkylamines and alkanethiols were restricted to the transfer of small nanoparticles [12,54]. Each method presented may only be able to facilitate the transfer of specifically modified nanoparticles. For this reason, it is desirable to create general phase transfer methods able to transfer a wide range of particles with different shapes and sizes.

Ligands that hold the ability to transfer particles < 10 nm often encounter complications for the transfer of larger particles [4]. Ionic interactions can limit particle concentration in aqueous synthesis. This is often overcome with low reaction concentrations or the addition of stabilizers [5].

Phase transfer of aqueous nanoparticles after synthesis is desirable because many of the available inexpensive metal precursors and reducing agents are water soluble [6]. Once synthesized, the transfer of particles into organic media allows for easier processing of the nanoparticle solutions [6]. The steric stabilization of nanoparticles can provide colloidal solutions that can be dried, purified and redissolved at higher concentrations. This helps the stability of the particles over long periods of storage [6].

An advantage of nanoparticle synthesis in organic media is the ability to synthesize particles at high concentration. The ionic interactions normally found in aqueous systems forcing low precursor concentrations are not present in organic media [6,30]. It would be beneficial to develop a theory that defines the properties needed for phase transfer for each different method. By establishing the required criteria for phase transfer, new methods and materials can be identified.

Current procedures for the phase transfer of nanoparticles are limited in the types and sizes of nanoparticles that can be effectively transferred. There is a general trend in the literature where lengthening alkyl chains is important for the transfer of large nanoparticles [5,8,14]. Without the long alkyl chain, the ligands often cannot fully stabilize large particles. The phase transfer of larger particles has been carried out by extending the chain length on ligands that can transfer smaller particles [8]. Many techniques for phase transfer are still being discovered. Finding ligands that have properties to facilitate pH-triggered, host–guest, and thermoregulated transfer are of much interest. These methods allow for quick and effective transfer of particles by modifying the chemical environment, rather than the particles themselves [35]. The biggest obstacle of nanoparticle phase transfer is retaining the physical and chemical properties, as well as the stability of nanoparticles after the transfer.

## 5. Applications of Phase Transfer in Nanochemistry

Nanoparticles have found use in applications such as drug carriers for targeted delivery and controlled release of therapeutic cargo [2,29,30,61,62]. Nanoparticles can offer many unique properties in drug delivery systems offers many such as decreased side effects, dose, and dose frequency [2]. The solubility of nanoparticles often poses problems for the loading and unloading of drug cargo [61]. The transfer of stabilized nanoparticles from a polar to non-polar environment is often required to account for this phase barrier [2]. The phase transfer of nanoparticles is generally carried out through alteration of the hydrophilicity of the particle surface [2]. Understanding the physiochemical properties of nanoparticles and their diffusion in a biological system is important for improving the efficiency and safety of nanoparticles for use in drug delivery.

NPs made of different materials, shapes, and sizes are often synthesized in aqueous solutions and transferred to the desired phase via phase transfer protocols which remove solubility barrier and allow reactions to proceed. The stability of NPs in aqueous media is of extreme importance when they used in biological applications [2]. The use of amphiphilic polymers to stabilize nanoparticles produces useful systems for biologically relevant applications [2].

Phase transfer is generally a required step to stabilize hydrophobic NPs as their isolation in a pure state is not easily achieved [2]. Various techniques, as described above, have been used to transfer the organic phase nanoparticles into water, specifically aimed for their use in biological systems [2,29].

The high chemical stability of gold nanoparticles make them suitable for exploring many phase transfer agents. In the study performed by Pablo del Pino and his group, spherical and rod Au nanoparticles were phase transferred with different sizes (diameters of 25, 50, and 60 nm up to nanorods with 90 nm in length) from an aqueous solution to an organic medium through the use of aliphatic chains with terminal thiol or amine groups [2]. DDA was used to facilitate the transfer of PEG particles [2]. The amphiphilic polymer coating enables the particles to be stable for use in harsh biological conditions with high protein concentrations [2].

Another study, conducted by Wang and his group, demonstrated that the phase transfer of gold nanoparticles of different sizes and shapes from an aqueous to organic solution can be accomplished using an ionic liquid medium [55]. In this case, a cationic surfactant was used to transfer gold nanoparticles to organic phases [45]. One positive trait of this method is that is does not require surface modification of the precursor nanoparticles to enable transfer. This method permits one to transfer nanoparticles to a desired solvent while maintaining chemical and physical properties.

Phase transfer of metallic nanoparticles can also prove useful for use in catalytic systems for the separation of nanoparticles from the reaction system [63]. Metallic nanoparticles as catalysts are of much interest due to their unique properties and high efficacy. One of the toughest problems with using soluble nanoparticle catalysts is the separation of the catalyst from the reactants and products [35]. Traditional methods cannot be used because they can contaminate the final product in solution. That is why the systems which are amenable for recovery and redispersion of catalytic nanoparticles that retain their shape, size, and reactivity are highly desired. The ability to recycle nanoparticles without reduction in catalytic activity would reduce costs and increase the efficiency of the reaction. Many phase transfer methods to recover nanoparticle catalysts involve pH-dependent, host–guest, or thermoregulated transfer, as described in previous sections [3,35,39]. A common theme in these types of phase transfer is aggregation of particles followed by redispersion in separate phases. These methods provide phase transfer and recovery of nanoparticles without altering or affecting the reaction being catalyzed.

For example, in the study reported by Chakraborty and Kitchens, thiolated pH-responsive PAA-SH ligand was synthesized and used to functionalize efficiently reusable colloidal AuNP catalysts [3]. They found that catalytic activity and recoverability depends on the ligand chemistry and the surface packing density [3]. PAA-SH has a higher molecular weight and therefore a lower packing density on the AuNPs surface [3].

Phase transfer methods that utilize pH-responsiveness are of great importance because they circumvent the multistep extractions and resulting degradation of nanoparticles in catalysis. These pH-triggered transfer methods allow for nanoparticles to be recovered without affecting the reactants or product formation. Using a thermoregulated phase transfer system is another way to overcome this problem. Thermoregulated phase transfer catalysis (TRPTC) is the process of changing the temperature of the system to separate the catalyst from the product in the reaction mixture. In TRPTC, the temperature is first lowered to stabilize the nanoparticle with the thermoregulated ligand, then the system is heated causing a change in the solubility of the nanoparticle, effectively transferring to an immiscible phase. This allows for the catalyst and substrate to be present in the same phase and the reaction can proceed homogenously. When the reaction is complete, the system is cooled in order to separate the catalyst from the product [41].

## 6. Conclusions

Phase transfer of nanoparticles can often be necessary for biochemical and drug delivery applications [2,29,30,61]. Particles for drug delivery must be soluble in aqueous media, as they must be stable throughout the human body [2]. However, many drugs are hydrophobic and therefore are not soluble in water. Phase transfer methods can bridge the gap and allow for nanoparticles to be loaded with a drug in one phase and be delivered in another phase. Phase transfer strategies allow for researchers to modify and load molecules onto particles that were tailored to a desired system. This is a beneficial option compared to utilizing specific particles that are stable in the reacting media directly after synthesis. 

The phase transfer of nanoparticles allows for the utilization of a wide variety of particles in applications which were previously limited due to solubility issues [64,65]. New phase transfer agents and methods continue to advance the use of nanoparticles in applications such as catalysis and drug delivery. Preserving the size, shape, and chemical properties of the transferred nanoparticles is of great importance. Further investigation into particle modification for phase transfer is needed to develop a general phase transfer method capable of transferring a wide variety of nanoparticles. 

## 7. Future Directions

Phase transfer techniques can provide insights into potentially new chemical properties and provide an opportunity for new applications of nanoparticles. Just as there are many well-defined methods for the synthesis of a variety of nanoparticles, there is a need for novel, effective, and diverse phase transfer techniques. Our interest in the area of phase transfer is need based. In our laboratory, we have developed new methodologies for one pot creation of novel metal nanoparticles in organic and aqueous solvents [66,67,68,69]. In our ongoing projects, we have water-soluble nanoparticles, which we want to tailor with drug molecules. However, the drug molecules of interest are only soluble in organic solvent. In order for the drug molecules to be loaded onto nanoparticles, we began looking at alternatives. This led us to investigate the phase transfer of nanoparticles created in our laboratory from aqueous to organic media. Currently, in our laboratory, we are in the process of finalizing the experimental conditions for transfer of silicon-containing metal nanoparticles from aqueous to organic phase. The results of these investigations will be published in due course.

Nanoparticle phase transfer methods may also prove useful in applications with nanofilms. In an example from Feng and collaborators, the smaller chain length of the transfer ligand allowed for nanoparticle phase transfer and sedimentation into nanofilms of various shapes and sizes [4]. The formation of single-crystalline gold nanoprisms is generally a slow process; however, through the application of modified phase transfer procedures, the yield can be greatly increased [4]. Other areas of future development could be where interdigitation, thermoregulation, and pH-triggered transfer can become more common phase transfer tools.

## Figures and Tables

**Table 1:** Representative methods for phase transfer summarized in this review.

Particle Type	Transfer Method	Phase Transfer Molecules	Particle Size Transferred (nm)	Solvent System	Reference
Au, Ag, Pd, Pt, Ru, TiO_2_,Alumina, CdSe, CdS, CdSe, PbS, ZnS	Ligand Exchange	Lipoic acid, UV irradiation	5 to 13	Organic to Aqueous	Ref. [41] and all those cited within
		Dioctylamine, didodecylamine, dioctadecylamine	5, 9, 13	Aqueous to Organic	Ref. [8] and all those cited within
		(Z)-octadec-9-en-1-yl-5-(1,2-dithiolan-3-yl)pentanoate	5 to 70	Aqueous to Organic	Ref. [11] and all those cited within
		*n*-propyl, *n*-pentyl, *n*-octyl, *n*-dodecyl, *n*-octadecyl, tert-butyl, andphenyl-phosphonic acid	4 to 8	Aqueous to Organic	Ref. [10] and all those cited within
		Thiolated polystyrene	3 to >100	Aqueous to Organic	Ref. [6] and all those cited within
		Dicyclohexylamine, mercaptoacetic acid	45 to 100	Aqueous to Organic	Ref. [12] and all those cited within
		α-trithiocarbonate-ω-carboxyl-terminated poly(*N*-isopropylacrylamide)	15 to 53	Aqueous to Organic	Ref. [13] and all those cited within
		Mercaptophenol, mercaptotoluene, propanethiol	1.5	Organic to Aqueous	Ref. [15] and all those cited within
		1-Decanethiol, 1-dodecanethiol, 1-tetradecanethiol	5	Aqueous to Organic	Ref. [18] and all those cited within
		Thiolated poly(ethylene glycol), dodecanethiol	up to 200	Aqueous to Organic	Ref. [19] and all those cited within
		Tetraoctylammonium bromide, hexadecyltrimethylammonium bromide	up to 16	Aqueous to Organic	Ref. [42] and all those cited within
		L-histidine, dihydrolipoic acid	3	Organic to Aqueous	Ref. [43] and all those cited within
		Cationic tetraoctammonium	5	Aqueous to Organic	Ref. [20] and all those cited within
		Octadecylmine, Dodecylamine	6, 12	Aqueous to Organic	Ref. [21] and all those cited within
		Dodecylamine, glutathione, tetramethylammonium salt	12.2, 7.6, 13.4	Aqueous to Organic/Organic to Aqueous	Ref. [22] and all those cited within
		Thiol-terminated poly(styrene), oleylamine	6.4, 12.3, 23.1, 40.0, 46.8, 61.3, 79.6, 89.8	Aqueous to Organic	Ref. [23] and all those cited within
		Dodecylamine	4	Aqueous to Organic	Ref. [24] and all those cited within
		Heptamine β-cyclodextrin, octylamine, oleic acid	3, 4	Organic to Aqueous	Ref. [25] and all those cited within
		Oleyl phosphate	5 to 20	Organic to Aqueous	Ref. [44] and all those cited within
		Glutathione tetramethylammonium salt (GTMA), tetraoctylammonium bromide (TOAB), CTAB	7.2 to 8.1	Organic to Aqueous/Aqueous to Organic	Ref. [45] and all those cited within
		Dodecylamine	3.45, 4.33, 7.89	Aqueous to Organic	Ref. [46] and all those cited within
		16-mercapto-N-octadecylhexadecanamide	13, 20	Aqueous to Organic	Ref. [47] and all those cited within
		Ocylamine, DDA, HDA, and ODA	5 to 37	Aqueous to Organic	Ref. [48] and all those cited within
		C-undecylcalix [4]-resorcinarene (C11-resorcinarene)	12	Aqueous to Organic	Ref. [49] and all those within
Au, Silica	pH-Triggered	Thiolated polyacrylic acid, octadecylamine, HCl	13	Aqueous to Organic	Ref. [3] and all those cited within
		Cetyltrimethylammonium chloride, tetramethyl orthosilicate, HCl, NaOH	200 to 300	Aqueous to Organic/Organic to Aqueous	Ref. [50] and all those cited within
Au, Ag, Fe_3_O_4_	Oleic Acid Modification	2-Aminoethanethiol modified oleic acid	13, 57	Aqueous to Organic	Ref. [51] and all those cited within
		Oleic acid, ammonium hydroxide	12	Aqueous to Organic	Ref. [28] and all those cited within
Au, Fe_3_O_4_, Silica	Polymer Systems	Polyethylene glycol, dodecylamine, polyisobutylene maleic anhydride	25, 50, 60, 90	Aqueous to Organic	Ref. [2] and all those cited within
		Poly(vinylpyrrolidone)	14	Organic to Aqueous	Ref. [29] and all those cited within
		Poly(methylmethacrylate), poly(vinylacetate)	15	Aqueous to Organic/Organic to Aqueous	Ref. [32] and all those cited within
		Methacrylated spiropyran	400	Aqueous to Organic/Organic to Aqueous	Ref. [36] and all those cited within
Au, Ag, Fe_3_O_4_	Host–guest	α-cyclodextrin, oleic acid	8, 10	Organic to Aqueous	Ref. [27] and all those cited within
		2-Hydroxypropyl)-β-cyclodextrin	15	Organic to Aqueous	Ref. [52] and all those cited within
		per-6-Thio-α-cyclodextrin, (dimethylaminomethyl)azobenzene	3.6	Aqueous to Organic/Organic to Aqueous	Ref. [35] and all those cited within
		Alkyldimethyl(ferrocenylmethyl)ammonium ions	3	Aqueous to Organic	Ref. [53] and all those cited within
		Tetreaoctylammonium bromide, mercaptosuccinic acid	3	Aqueous to Organic	Ref. [54] and all those cited within
		Butyl-3-methylimidazolium hexafluorophosphate	48	Aqueous to Organic	Ref. [55] and all those cited within
Au, Ag, Fe_3_O_4_, C_60_	Janus Particles	Aptamer-pendant DNA tetrahedron	58	Organic to Aqueous	Ref. [56] and all those cited within
		3-Mercapto-1,2-propanediol, hexanethiolate	5, 7.4	Aqueous to Organic/Organic to Aqueous	Ref. [38] and all those cited within
Au	Interdigitated Layers	Cetyltrimethylammonium bromide, dodecylamine,	16	Organic to Aqueous	Ref. [7] and all those cited within
Au, Ag, Pt, CdTe	Thermoregulated	Poly(*N*-vinylpyrrolidone), heat at 80 °C	35	Aqueous to Organic	Ref. [4] and all those cited within
		Poly(*N*-isopropylacrylamide)	7	Aqueous to Organic/Organic to Aqueous	Ref. [31] and all those cited within
		2-(Diethylamino)ethanethiol	2.5, 3	Aqueous to Organic/Organic to Aqueous	Ref. [39] and all those cited within

## Data Availability

Not applicable.
